# Availability of drone mission with binary decision diagram based on uncertain data

**DOI:** 10.1038/s41598-026-42988-w

**Published:** 2026-03-14

**Authors:** Elena Zaitseva, Jan Rabcan, Vitaly Levashenko, Ravil I. Mukhamediev, Nicolae Brinzei

**Affiliations:** 1 Zilinska Univerzita v Ziline, Zilina, 01026 Slovakia; 2 Institute of Automation and Information Technology, Satbayev University (KazNRTU), Almaty, 050013 Kazakhstan; 3 Université de Lorraine, CNRS, CRAN, Nancy, F-5400 France

**Keywords:** Drone, Unmanned aerial vehicle (UAV), Binary decision diagram (BDD), Fuzzy decision tree, Reliability analysis, Availability, Importance measures, Engineering, Mathematics and computing

## Abstract

Unmanned aerial vehicles (UAVs), or drones, are increasingly deployed for critical missions such as environmental monitoring, infrastructure inspection, and disaster response. Assessing the reliability of these missions is essential for operational planning, yet conventional approaches often fail when input data are incomplete or epistemically uncertain. We present a novel framework for mission availability analysis that integrates fuzzy decision tree (FDT) induction with binary decision diagram (BDD) construction. The method interprets a drone mission as a reliability system, where checkpoints act as components and mission success is modeled by a structure function. Expert evaluations expressed as confidence degrees are used to induce an FDT, which is subsequently defuzzified and transformed into a canonical BDD. This representation enables efficient computation of mission availability and sensitivity measures using established BDD algorithms. We validate the approach on a real-world case study of a forest fire monitoring mission comprising eight checkpoints and demonstrate high predictive accuracy (94%) despite incomplete training data. The proposed method provides a transparent, reproducible pipeline for translating uncertain, expert-driven data into quantitative reliability metrics, offering practical insights for mission planning under uncertainty.

## Introduction

In recent years, the industrial application of drones has expanded significantly due to their relatively low cost, versatility, and strong support from both industrial and scientific sectors. Unmanned Aerial Vehicles (UAVs), or drones, have transitioned from niche technologies to core components in critical civilian and industrial missions, including environmental monitoring, infrastructure inspection, precision agriculture, and disaster response^1,2^. The success of these missions depends not only on the hardware reliability of individual drones but, more critically, on the operational success of the mission as a whole, which is a complex system where task completion, data quality, and environmental factors intersect^3,4^. The operational success of the mission, however, is often threatened by uncertain and incomplete data regarding the reliability of individual mission segments, which can be considered epistemic uncertainty^5,6^. Traditional reliability engineering methods, which require precise probabilistic data, fall short in this context, leaving mission planners without quantitative tools to assess the likelihood of mission success^7,8^.

Recent advances in uncertainty modelling, such as evidence theory and fuzzy logic, have been applied to system reliability^9,10^, but they often require bespoke computational frameworks. A pragmatic alternative is to transform uncertain data into standard reliability models, thereby leveraging established, efficient analysis algorithms^11^. The Binary Decision Diagram (BDD) is one such model that provides a graph-based representation of system logic, enabling the rapid computation of system availability^12,13^. BDD is an effective representation of a large-dimensional structure function in reliability analysis. A structure function is a mathematical model of a system that maps the sets of all possible component states to the system state^14,15^.

In prior work, we developed a general method to construct BDDs from epistemically uncertain data using Fuzzy Decision Trees (FDTs) as classifiers^13^. Here, we specialise and apply this framework to a novel domain: drone mission availability analysis. We formalise a monitoring mission as a system for reliability analysis, based on a set of spatially distributed checkpoints (interpreted as system components). Expert evaluations of checkpoint performance, expressed as confidence degrees, are used to induce an FDT, which is then transformed into a BDD representing the mission’s success logic.

The core contribution of this study is a data-driven, quantitative framework for mission availability assessment under epistemic uncertainty. We demonstrate its utility through a real-world case study: a drone-based forest fire monitoring mission with eight checkpoints. The resulting BDD model allows us to compute the mission’s availability.

This work bridges the gap between high-level mission planning and low-level reliability analysis, providing a transparent, computable model that translates uncertain, expert-driven data into a concrete probability of mission success. A new method is proposed for the quantitative assessment of drone-based monitoring missions’ availability, employing well-known algorithms for FDT induction, BDD construction, and BDD-based calculation of reliability metrics. By focusing on the mission as the system of interest and providing a direct path from uncertain, real-world data to a quantitative availability metric, this work moves beyond generic reliability analysis. It offers a practical tool for engineers and planners to assess and improve the success probability of complex drone operations in the face of incomplete knowledge. Our core contribution is threefold:

1. Mission formalisation in terms of the reliability analysis domain. We interpret a drone monitoring mission as a system whose operation depends on some components (checkpoints or segments of a path). Mission success is modelled by a structure function, where component states represent the successful data acquisition at each checkpoint, often evaluated with expert confidence degrees.
2. Methodology adaptation and application of the FDT-to-BDD construction method to handle mission-specific data, characterised by epistemic uncertainty from expert evaluations and partial observations. The resulting BDD serves as a transparent, computable model of the mission’s success logic.
3. Mission-Centric Analysis based on the constructed BDD to compute the probability of mission success, that is, mission availability.

## Result for analysis of forest fire monitoring mission

### Binary decision diagram model of the forest fire monitoring mission

Consider the drone mission for monitoring of forest fires. The goal of this monitoring is to collect data from 8 checkpoints (*x*_1_, *x*_2_, …, *x*_8_). The mission succeeds only if a specific logical combination of checkpoints provides sufficient data for assessing fire risk. The full dataset for developing a mathematical model to evaluate the mission’s availability must contain 256 instances. 150 observations (instances) were provided for this mission evaluation (the provided data is in the table form https://www.dropbox.com/scl/fi/ck0jnl4btcqp675xa22vl/Mission-02.xlsx?rlkey=xe5muebh2trfnygkzqezuutc3&dl=0). Each instance includes the confidence of value (0 or 1) of data applicability from checkpoint *i* (*i* = 1, …, 8) and two values of confidence for the mission result (0 if the prognosis is not possible and 1, if the prognosis is done). For example, checkpoint *i* has a value of 0 with a confidence of 0.1 and a value of 1 with a confidence of 0.9. It means that data from this checkpoint can be applicable, but it may have some minor inaccuracies.

**Fig. 1:**
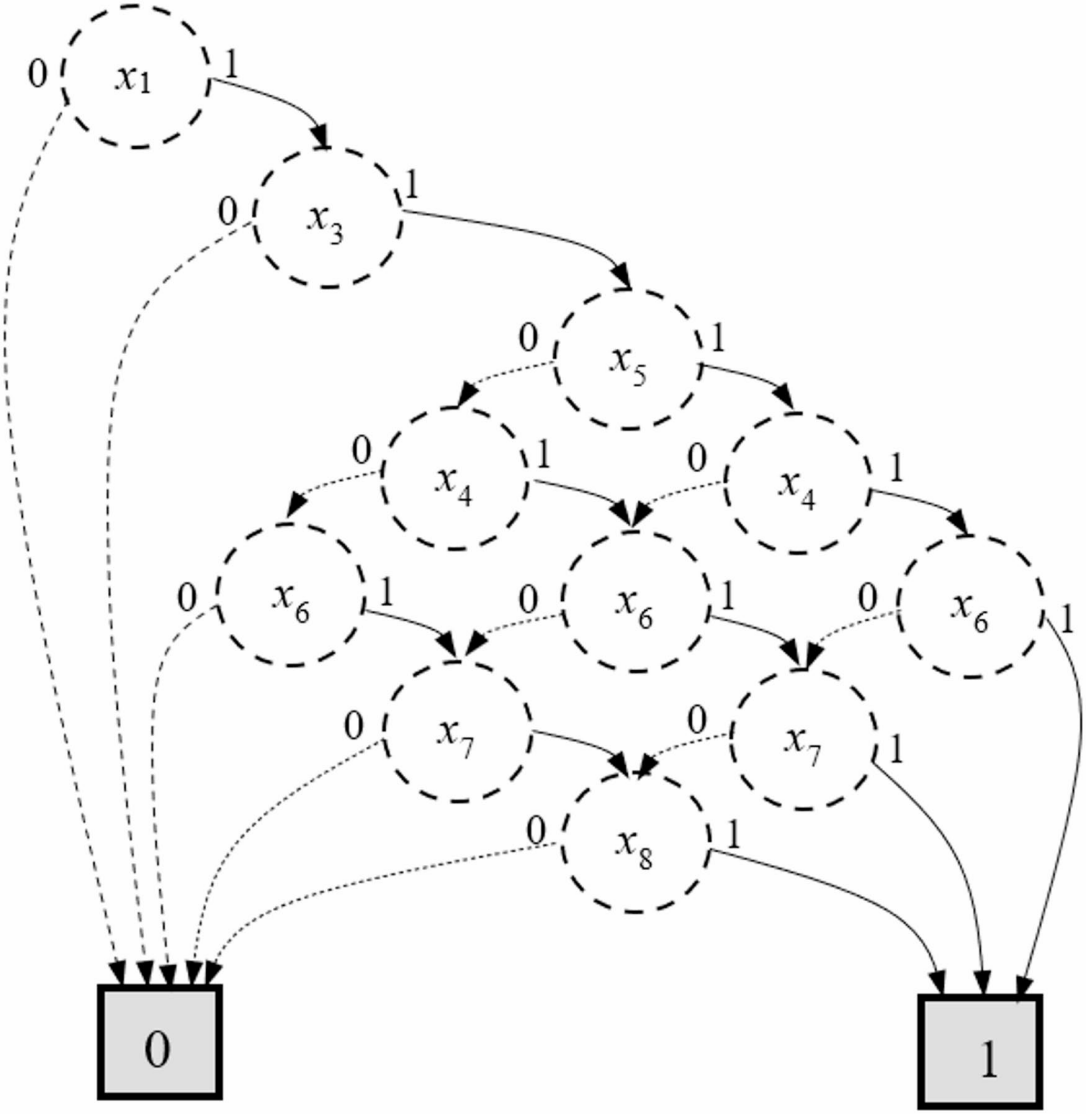
The BDD of the structure function of the mission for terrene forest fire monitoring.

Using the proposed method on the dataset of 150 expert evaluations (see Methods), the BDD (Fig. 1) is constructed through the FDT induction. The BDD compactly encodes the mission success logic, with non-terminal nodes corresponding to checkpoint states and terminal nodes indicating mission success (1) or failure (0). The structure reveals, for instance, that the checkpoint ${x}_{1}$acts as a logical bottleneck, because if it fails, the mission fails regardless of the states of other checkpoints. Nodes represent checkpoints ${x}_{i}$, solid edges indicate successful data acquisition (${x}_{i}=1$), dashed edges indicate failure (${x}_{i}=0$). Terminal node ‘1’ denotes mission success, ‘0’ denotes failure.

### Mission analysis

Using the BDD model and checkpoint estimates derived from confidence degrees, the probability of the mission’s success is computed. This probability refers to mission availability in the context of reliability analysis. The availability is computed as the sum of probabilities of all paths leading to the terminal node ‘1’. The mission availability is *A* = 0.992. This high availability indicates that, given the current estimate of probabilities of successful checkpoint passing, the mission is highly likely to succeed. The individual checkpoint success probabilities ${p}_{i}$ are listed in Table 1. These probabilities for this mission had been defined by experts.

**Table 1:** The probabilities of checkpoints’ states and the availability/unavailability of the mission.

	*x*_1_	*x*_2_	*x*_3_	*x*_4_	*x*_5_	*x*_6_	*x*_7_	*x*_8_
*p*_*i*_	0.90	0.79	0.930	0.860	0.860	0.860	0.720	0.720

To support mission planning, we evaluated how targeted improvements in specific checkpoints could enhance mission availability. Two practical scenarios were considered:

1. **Scenario A**: Improve the most sensitive checkpoint ${x}_{1}$from ${p}_{1}=0.90$ to ${p}_{1}=0.95$.
2. **Scenario B**: Improve the two most sensitive checkpoints ${x}_{1}$and ${x}_{2}$to ${p}_{1}=0.95,{p}_{2}=0.90$.

The results are summarised in Table 2.

**Table 2:** Mission availability under improvement scenarios.

Scenario	Checkpoint successful probability changes	Resulting availability
Baseline	–	0.992
A	${p}_{1}=0.90$→${p}_{1}=0.95$	0.996
B	${p}_{1}=0.90$→${p}_{1}=0.95$ and ${p}_{2}=0.79$ → ${p}_{2}=0.90$	0.998

Even modest improvements in the most critical checkpoints lead to a measurable increase in mission availability, with Scenario B achieving $A=0.998$.

### Robustness to data incompleteness

Given that the training dataset contained only 150 out of 256 possible state vectors (~ 58% completeness), we evaluated the robustness of the induced BDD. The model was tested on a held-out validation set of 50 expert evaluations not used in training. The BDD correctly predicted mission success/failure with 94% accuracy, demonstrating that the method yields a reliable mission logic model even with partially specified data.

## Preliminaries: reliability analysis of drone mission

A drone mission consists of one or more tasks that the drone(s) should perform to achieve the mission goal. In monitoring, these tasks correspond to the passage of specified segments of the drone’s path or predefined checkpoints. From a reliability analysis perspective, these specified segments or checkpoints can be viewed as system components. The successful implementation of the mission is interpreted as the system’s successful functioning, and the mission’s failure is considered a failure of the system. In some studies, such systems are considered phased mission systems^16^. Considering the successful monitoring of each specified segment/checkpoint as an independent event allows us to interpret the drone mission as a system in a stationary state.

**Fig. 2:**
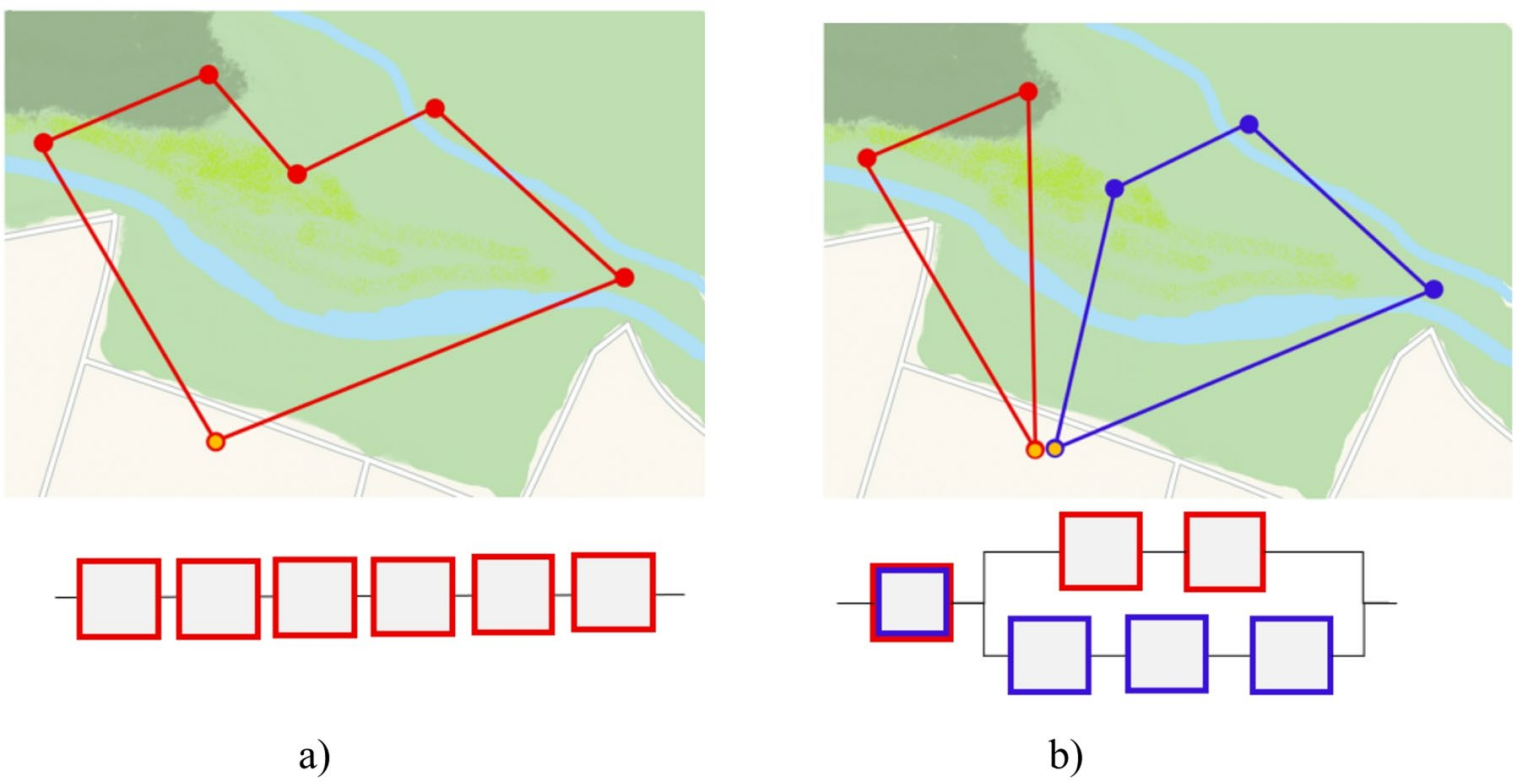
The interpretation of the drone mission by reliability block diagrams for one drone (**a**) and two drones (**b**) (*Figure created by the authors as an OSM‑style schematic map; no external copyrighted material was used*).

For example, let’s assume a monitoring mission covers 7 checkpoints (Fig. 2). This mission can be implemented by a single drone (Fig. 2, a), and in this case, the system is represented as a series system from a reliability analysis perspective. The simultaneous use of two drones for this mission allows the representation of this mission as a series-parallel system (Fig. 2, b). Both of these interpretations can be analysed based on typical reliability methods.

A mathematical model of a drone mission must be developed to analyse and evaluate its reliability. In the context of UAV operations, the terms mission reliability, mission availability, and mission success probability are often used interchangeably, although they represent related but distinct concepts. The probability of mission success may be defined empirically as the number of successfully completed missions divided by the total number of missions. This empirical measure reflects observed mission performance and, by definition, does not require information about the success or failure of individual path segments or checkpoints. Mission reliability is traditionally defined for time‑dependent systems as the probability of completing a mission without failure over a specified mission duration. When the mission is executed once and no repair during the mission is possible (as is typical for UAV operations), mission reliability reduces to a one‑shot probability of successful completion. Mission availability characterises the probability that the system is in an operational state at an arbitrarily chosen point in time. In classical reliability engineering, this measure is time‑dependent and requires modelling failure and repair processes. In our study, however, availability is used in a static sense: it corresponds to the probability that components required for system (mission) operation are in the success state at the evaluation moment.

Many mathematical approaches (Markov model, Petri nets, algebra logic, and others) are used to construct a system’s mathematical model. This paper proposes a structural function for the reliability analysis of drone missions.

### Structure functions

Let us consider a monitoring drone mission involving *n* specified path segments or checkpoints, which is interpreted as a system of *n* components in the context of reliability analysis. These components can be in two states: functioning (the path segment or checkpoint is successfully passed) and failure (the path segment or checkpoint is not passed). A structure function of a monitoring mission defines its performance depending on the states of its components and is represented by a Boolean function^13,15^:

1 $$\phi({x_1}, \ldots ,{x_n})=\phi\left( x \right):{\left\{ {0,1} \right\}^n} \to \left\{ {0,1} \right\}$$

where the variable *x*_*i*_ (*i* = 1,…,*n*) represents the *i*-th system component states (segment of a path or checkpoint): *x*_*i*_ = 0 if the path’s segment or checkpoint is not passed (the component is failing) and *x*_*i*_ = 1 if the segment or checkpoint is successfully passed (the component is functioning); the function value $\phi$(***x***) represents the result of mission implementation: $\phi$(***x***) = 0 is the mission failure and $\phi$(***x***) = 1 is the mission successful; ***x*** = (*x*_1_,…, *x*_*n*_) is state vector of the mission.

**Fig. 3:**
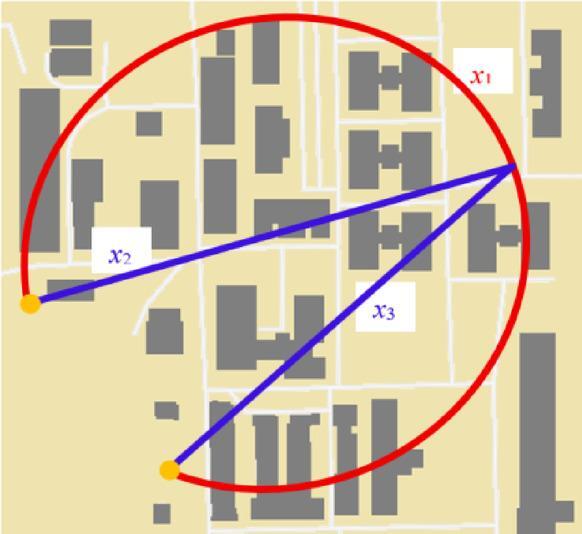
The monitoring mission of two drones (*Figure created by the authors as an OSM‑style schematic map; no external copyrighted material was used*).

For example, the monitoring drone-based system consists of two drones (Fig. 3). One of the drones has only one segment for control (*x*_1_) and another drone monitors two segments (*x*_2_, *x*_3_). This mission is successful if one of these drones implements its monitoring. The system has three components (*n* = 3):

2 $$\phi\left( x \right){\text{ }}={x_1} \vee ({x_2} \wedge {x_3})$$

where ⋀ and ⋁ are operators of conjunction and disjunction, respectively.

The structure function for reliability analysis can be represented by a Boolean expression (1), the truth table (Table 1), a reliability block diagram, a fault tree or other. However, not all of these representations are suitable for systems of large dimensions. Another representation of a structure function for large dimensions in reliability analysis is the BDD^13,14,17^.

One more advantage of a structure function is the representation of redundancy of a mission. It can be defined using the typical *k*-out-of-*n* system as a mathematical model, which is often used to represent drone mission behaviour^7,16^. For example, let there be a mission with 3 checkpoints; it will be successful if information from 2 of them is collected. It means that one of these checkpoints is redundant. It means that one of these checkpoints is redundant. The truth table of this mission’s structure function is in Table 3.

**Table 3:** The Example of the Structure Function of the Redundant Mission System (2-out-3).

*x*_1_	*x*_2_	*x*_3_	*ϕ*(*x*)
0	0	0	0
0	0	1	0
0	1	0	0
0	1	1	1
1	0	0	1
1	0	1	1
1	1	0	1
1	1	1	1

### Binary decision diagram

S.B.Akers introduced BDD as a graphical representation of the process of a Boolean function of a large dimension^18^. Later, this mathematical representation of Boolean functions was used in reliability analysis for the structural function of the system^15^. The background of BDD is the Shannon expansion for Boolean functions^17^:

3 $$\phi\left( x \right){\text{ }}={\text{ }}(0 \wedge \phi\left( {{0_i},x} \right)) \vee (1 \wedge \phi \left( {{1_i},x} \right))$$

where $$\phi$$(*s*_*i*_, ***x***)= $$\phi$$(*x*_1_, …, *x*_(*i*−1)_, *s*, *x*_(*i*+1)_, *x*_*n*_) for *s* ∈ {0, 1}.

The Shannon expansion (3) allows a definition of format if-then-else (*ite*) for BDD manipulation: *ite*(a, b_0_, b_1_) = if a then b_0_, else b_1_. This property of the Shannon expansion reduces the computational complexity of the BDD-based representation of the structure function (1).

BDD is a tree-based graphical structure: the terminal nodes are system states and labelled 0 and 1; non-terminal nodes are associated with component states. Each non-terminal node is labelled by variables *x*_*i*_ (*i* = 1, …, *n*) and has 2 outgoing edges, which correspond to component states. There are two types of BDD, which are ordered BDD and no-ordered BDD. In an ordered BDD, levels can be specified, and each level is formed by a single variable. This property is not in a no-ordered BDD.

**Fig. 4:**
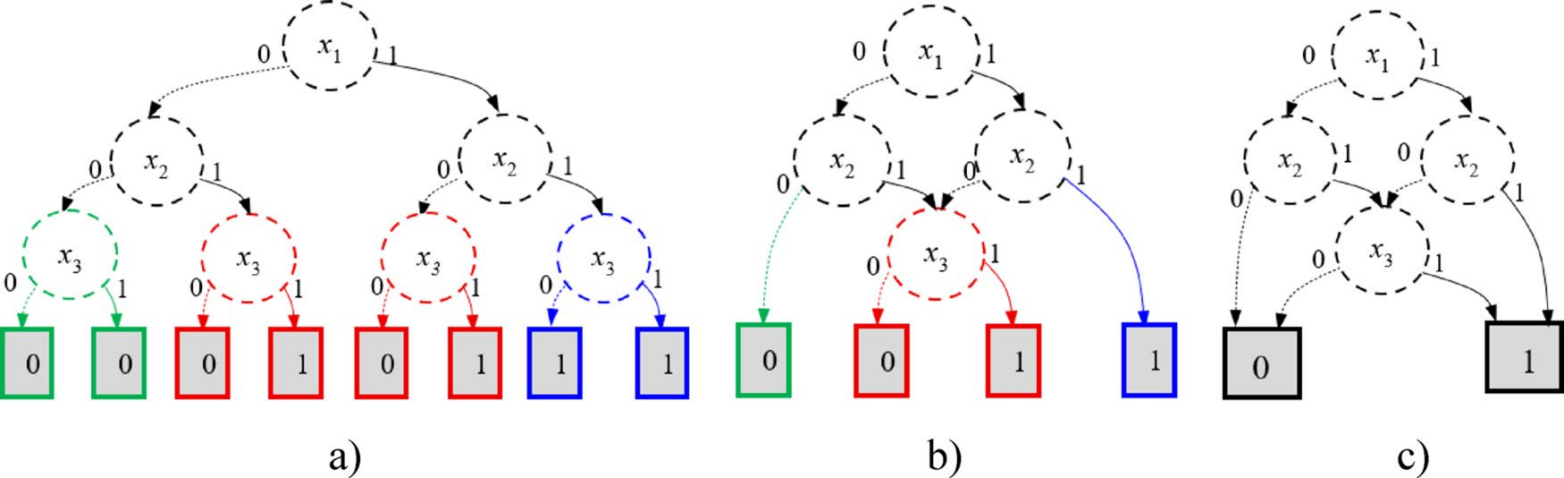
The BDD construction for the structure function of redundant mission from a decision tree (**a**), restricted structure (**b**) to BDD (**c**).

There are many algorithms for BDD construction. One of the basic algorithms was introduced in^18^, where BDD development is based on binary decision tree processing. The transformation of a decision tree into BDD is implemented using two rules: merging identical subgraphs, removing redundant (non-affecting) nodes, and merging identical sheets. For example, the BDD construction for the structure function of the redundant mission, interpreted as a 2-out-of-3 system (Table 3), is shown in Fig. 4. Figure 4(a) shows the decision tree of the structure function, developed according to the structure function in Table 3. This decision tree has two equal subgraphs, marked in red, and two non-affecting nodes, marked in green and blue. Therefore, the “red” subgraphs can be merged into a single subgraph, and the marked nodes can be removed (Fig. 4 (b)). The last step in constructing a BDD is merging identical leaves (Fig. 4(c)).

According to^18^, BDD is a canonical and orthogonal representation of a Boolean function. Therefore, the outgoing edges manipulation allows considering the probabilities of the *i*-th component state *p*_*i*_ (the probability of component *i* functioning) and *q*_*i*_ (the probability of component *i* fault):

4 $${p_i}={\text{ }}Pr\left\{ {{x_i}={\text{ }}1} \right\}{\text{ }} \mathrm{and} \;\;  {q_i}={\text{ }}Pr\left\{ {{x_i}={\text{ }}0} \right\}$$

In the context of monitoring mission availability analysis, the probabilities of component states (4) of successful or failed implementation of monitoring along the indicated path segments or checkpoints are defined by experts based on their knowledge of similar missions. In some missions, these probabilities can be defined based on observations, as considered in Sect.  4.2 below.

System availability *A* and unavailability *U* are computed based on the structure function:

5 $$A\,=\,Pr\{ \phi\left( x \right)\,=\,1\} {\text{ }} \mathrm{and} \;\; U\,=\,Pr\{ \phi\left( x \right)\,=\,0\}$$

**Fig. 5:**
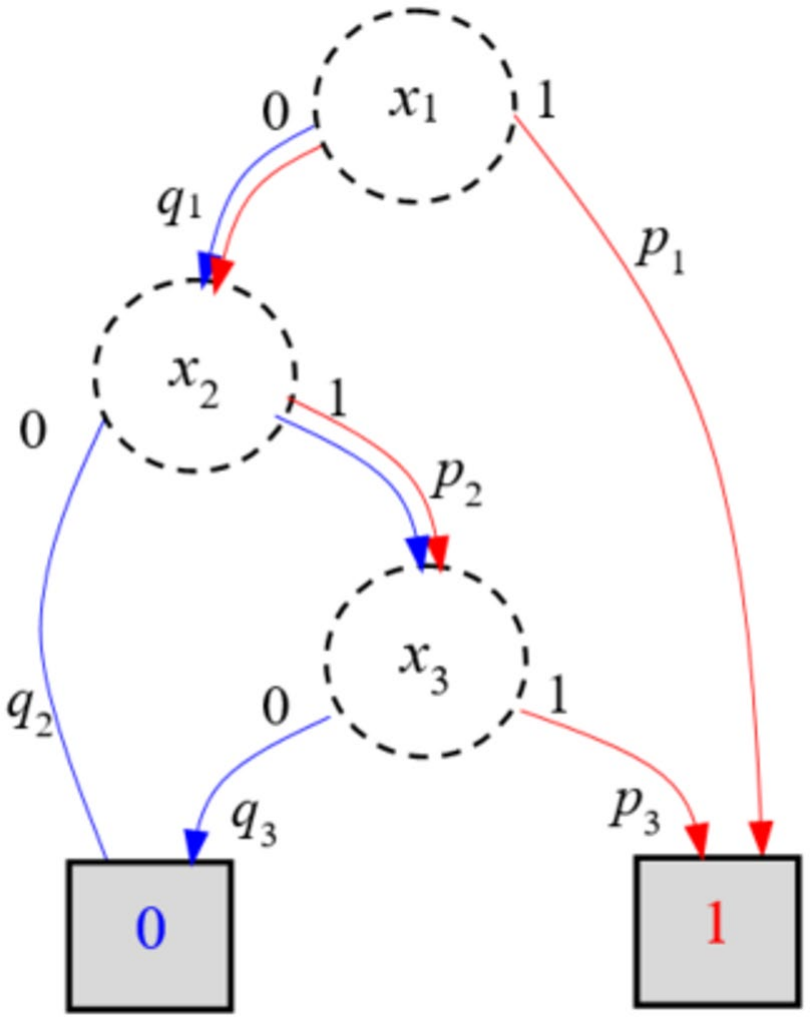
The BDD of the structure function of the mission system.

For example, the availability and unavailability of the mission system (Table 4) based on BDD (Fig. 5) are computed according to paths from the root node to the terminal nodes 1 and 0, respectively, and are given by *A* = *p*_1_ + *q*1 •* p*_2_ • *p*_*3*_ and *U* = *q*1 • (*q*_2_ + *p*_2_ • *q*_3_). For example, *A* = 0.962 and *U* = 0.038 if the probability of successfully monitoring the first drone is *p*_1_ = 0.8 and the two other segments by the second drone are *p*_2_ = 0.9 and *p*_3_ = 0.9.

**Table 4:** The Example of the Structure Function of the Mission System.

*x*_1_	*x*_2_	*x*_3_	*ϕ*(*x*)
0	0	0	0
0	0	1	0
0	1	0	0
0	1	1	1
1	0	0	0
1	0	1	1
1	1	0	1
1	1	1	1

Evaluating drone missions by their availability and unavailability is useful, but other reliability measures can also be beneficial. In particular, quantifying the impact of a drone failure in one of the specified monitoring segments on the mission’s overall success can be useful. It can be evaluated based on the importance analysis method.

## Data for reliability evaluation of drone mission

Data for drone mission reliability analysis originates from multiple sources, including historical mission logs, sensor outputs, and expert evaluations. This data can range from simple counts of successful task completions at specific path segments or checkpoints to complex metrics derived from big data analytics^2,19^. For instance, consider 10 observations of data collection at a fixed checkpoint, with 7 recorded as successful (e.g., 2 instances of data loss and 1 instance of the drone deviating from its path). This might yield an initial empirical success confidence of 0.7. However, an expert, considering contextual factors, might adjust this confidence to 0.75. In other scenarios, such as during the initial design or planning phase of a mission, no observational data may exist^20,21^. In such cases, confidence levels are derived solely from expert judgment.

Consequently, the data available for mission reliability assessment is often characterised by uncertainty—manifesting as vagueness, ambiguity, and incompleteness. This epistemic uncertainty renders traditional, deterministic reliability evaluation methods inadequate. Therefore, a new methodology is required to construct a system model, specifically a BDD, directly from such imperfect data.

The problem of BDD construction under data uncertainty is relatively under-explored. While it has been studied in logic design^22^, the interpretation of unspecified data differs fundamentally between domains. In logic design, an unspecified value in a Boolean function typically represents a “don’t-care” condition, referring to an input combination that is impossible or irrelevant, allowing for arbitrary assignment to optimise circuit design. In reliability analysis, an unspecified state of the structure function represents a gap in knowledge about the system’s behaviour for a given combination of component states. These values must be predicted or inferred based on the known portions of the function, as they correspond to plausible, yet unobserved, system conditions.

This distinction is critical. Applying logic design methodologies directly to reliability problems can lead to structurally incorrect BDDs that misrepresent the importance of components or system logic. Standard reliability methods also fail when data is not only incomplete but also vague (e.g., “confidence is high”) or ambiguous, which is best modelled using frameworks for epistemic uncertainty, such as fuzzy sets.

Given these challenges, we reframe the core problem: constructing a BDD from uncertain data is equivalent to inducing a binary classifier that partitions all possible state vectors of system components into two classes (system operational ($\phi$(***x***) = 1) and system failed ($\phi$(***x***) = 0)) based on incomplete and fuzzy evidence. This perspective allows us to leverage machine learning techniques for classifier induction from imperfect data.

Specifically, we propose using a FDT as the foundational classifier. An FDT is capable of processing data where attributes (component states) and/or class labels (system state) are associated with membership degrees (*µ*) in the interval [0, 1], representing confidence levels. The choice of a tree-based classifier is strategic, as a BDD itself is a canonical, directed acyclic graph derived from a decision tree structure. Therefore, transforming an induced FDT into an equivalent BDD is a natural and computationally efficient step. This approach provides a principled method for building a BDD-based mathematical model of a drone mission, enabling subsequent reliability and importance analysis even when the initial mission data are uncertain and incompletely specified.

## Method of drone mission evaluation

### Method for the availability calculation of the drone mission

The core of our approach for assessing drone mission availability is a method that constructs a BDD from uncertain and incomplete data. This method is a direct extension and application of the framework developed in our prior work^13^ for analysing the reliability of generic systems. We specifically adapt and refine this framework to address the unique challenges of modelling drone missions, where the success criteria depend on complex logical interactions (e.g., achieving coverage of specific checkpoints) rather than just component functionality, and where operational data is inherently vague or limited.

**Fig. 6:**
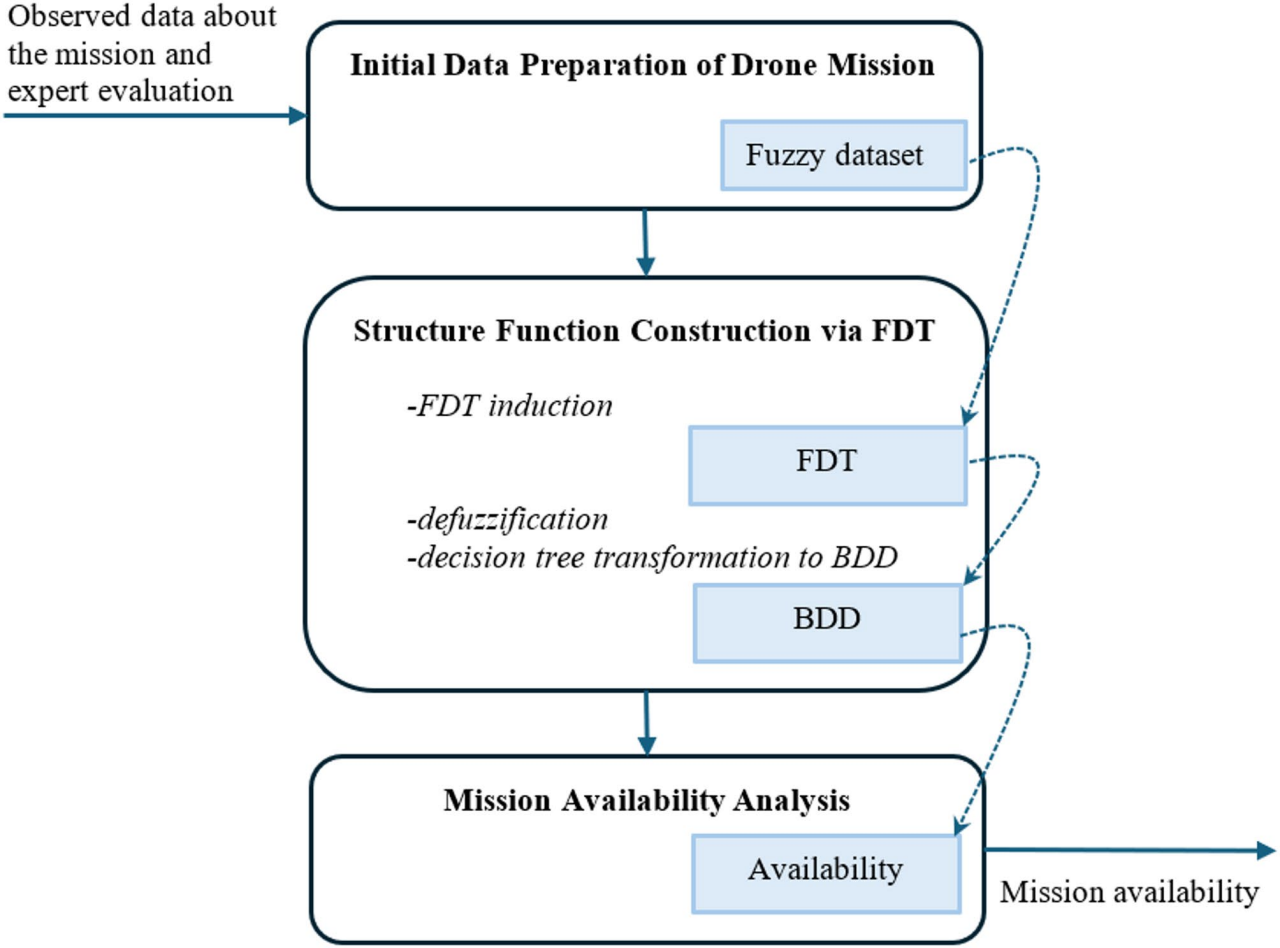
Principal stages of the proposed method for the reliability analysis based on BDD construction.

The proposed method for BDD construction and subsequent mission reliability analysis is illustrated in Fig. 6 and comprises three principal stages: (1) Initial Data Preparation, (2) Structure Function Construction, and (3) Reliability Analysis.

1. **Initial Data Preparation of Drone Mission.** This stage structures the raw data from mission logs, expert evaluations, and simulations into a form suitable for machine learning. For a drone mission with n critical checkpoints or functional elements, each is treated as a system component. Observations are recorded as instances where each component state (data successfully acquired = 1, failed = 0) and the overall mission state (success = 1, failure = 0) are annotated. Every instance includes the values of *n* input attributes A_1_, …, A_*n*_ and the value of the output attribute B, or, in other words, each instance includes an evaluation of each segment/checkpoint of the drone’s path and the mission evaluation. There are two values for the input attributes and the output attribute: A_*i*,0_ and A_*i*,1_ for the A_*i*_ input attribute, and B_0_ or B_1_ for the output attribute. Key to handling epistemic uncertainty, the “state” of a component (e.g., data quality at a checkpoint) is not treated as binary but as a *membership degree* within the interval [0, 1], thereby transforming the dataset into fuzzy data. If necessary, techniques such as fuzzification can be employed^23^.
2. **Structure Function Construction via FDT.** This is the central stage where we apply and adapt the method from^13^. It involves two key procedures:

- **FDT Induction**: We induce an FDT from the prepared fuzzy dataset. The FDT acts as a classifier that learns the logical relationship between checkpoint states and mission success from the uncertain data. For this, we employ the *Cumulative Mutual Information* (CMI)-based induction method detailed in^11^. This method selects the most informative component (checkpoint) at each node to build a tree that is robust to data incompleteness. Pruning parameters (α, β) control the tree’s depth and prevent overfitting, balancing model accuracy and generalisation.
- **Transformation to BDD**: The induced FDT is then converted into a BDD, the target mathematical model for efficient analysis. This conversion involves:
  1. **Defuzzification**: A crisp decision tree is obtained from the FDT by applying a mean-of-maximum (MOM) defuzzification rule to each leaf, assigning a definitive mission success (1) or failure (0) outcome.
  2. **BDD Generation**: The crisp decision tree is transformed and reduced into a canonical, minimal BDD using standard rules^18^: merging isomorphic subgraphs, deleting redundant nodes, and combining terminal nodes.

The choice of a tree-based classifier (FDT) is strategic and aligns with the core insight from^13^: the structural similarity between a decision tree and a BDD allows for a computationally simple and theoretically sound transformation. This step directly implements the methodological bridge between data-driven learning and formal reliability modelling established in our previous work.

**3. Mission Availability Analysis.** Once the BDD model of the mission logic is constructed, we compute key reliability metrics using established BDD algorithms^12–14,17^. This includes Mission Availability (*A*) or Unavailability (*U*), which is calculated by summing the probabilities of all paths in the BDD leading to the terminal node ‘1’ (success) or ‘0’ (failure), respectively, as defined in Eq. (5). The algorithms for these calculations based on BDDs are well-established and are directly applicable to the model generated by our method.

The primary contribution of this work is not the invention of a wholly new algorithm, but the targeted adaptation, integration, and application of an existing method from^13^ to a novel and critical domain: the quantitative reliability assessment of complex drone missions under epistemic uncertainty. While^13^ introduced the general framework of using an FDT to build a BDD from fuzzy data, the present study:

1. Interprets the drone mission as a reliability system, where components are mission-critical elements (e.g., checkpoints), and the system state is mission success.
2. Validates and demonstrates the method on specific, relevant case studies of drone fleets and monitoring missions, providing practical insights (e.g., which checkpoints are most critical).
3. Highlights the end-to-end process from fuzzy, mission-specific data to actionable availability and importance metrics, closing the gap between data uncertainty and operational decision-making for drone missions.

### Hand-calculation example for drone mission analysis

**Initial data preparation of a drone mission** availability analysis involves transforming and interpreting observations of segments of the path or checkpoints by the drone(s). These observations should be presented as a set of instances for FDT induction: each instance has values of *n* input attributes and the value of the output attribute. Most often, collected observations are represented in a table.

The use of a specific algorithm for FDT induction may impose some specific requirements on the data representation. For example, the CMI-based FDT induction algorithm mentioned^11^ requires that the sum of the two confidences of values of input attributes A_*i*,0_ and A_*i*,1_, or output attributes B_0_ and B_1_, equals one for each instance. At the same time, such an interpretation of the initial data allows us to determine the probability of success and failure for mission checkpoints or selected segments of the drone(s)’s path (4) not only based on expert knowledge but also taking into account observations. The probability (4) can be interpreted as the arithmetic mean of the values of each attribute:

6 $$p_i=\sum_{s}{A}_{i,1}/s \;\; \mathrm{a}\mathrm{n}\mathrm{d} \;\; q_i=\sum_{s}{A}_{i,0}/s$$

where *s* is the number of instances.

For example, the monitoring is based on the drone passing through 5 segments of the path. There are 13 observations for this mission (Table 5). Each instance of these observations has 5 input attributes that reflect whether the path’s segments were successful or not. The value in a cell of Table 5 is the confidence of the corresponding segment’s successful passing or not. The output attribute indicates whether the mission was successful or a failure, and its values are defined by the confidence of the success or failure of the mission. The last line in the Table introduces the interpretation of probabilities (6) for this example.

**Table 5:** The Initial Dataset for the System Analysis.

A_1_		A_2_		A_3_		A_4_		A_5_		B	
A_1,0_	A_1,1_	A_2,0_	A_2,1_	A_3,0_	A_3,1_	A_4,0_	A_4,1_	A_5,0_	A_5,1_	B_0_	B_1_
0.2	0.8	0.9	0.1	0.2	0.8	1.0	0.0	0.1	0.9	0.1	0.9
0.1	0.9	0.8	0.2	0.2	0.8	0.1	0.9	0.5	0.5	0.0	1.0
0.9	0.1	0.9	0.1	0.9	0.1	0.8	0.2	0.1	0.9	0.9	0.1
1.0	0.0	0.9	0.1	0.2	0.8	1.0	0.0	0.1	0.9	1.0	0.0
0.9	0.1	1.0	0.0	0.0	1.0	0.0	1.0	1	0	0.8	0.2
0.8	0.2	1.0	0.0	0.0	1.0	0.0	1.0	0	1	1.0	0.0
0.0	1.0	0.9	0.1	0.9	0.1	0.9	0.1	0.1	0.9	0.9	0.1
0.0	1.0	0.8	0.2	0.9	0.1	0.1	0.9	0.6	0.4	0.0	1.0
0.0	1.0	1.0	0.0	0.8	0.2	0.1	0.9	0	1	0.0	1.0
0.1	0.9	0.8	0.2	0.2	0.8	1.0	0.0	0.9	0.1	1.0	0.0
0.1	0.9	0.2	0.8	0.8	0.2	0.2	0.8	0.1	0.9	0.1	0.9
0.1	0.9	0.0	1.0	0.1	0.9	0.8	0.2	1	0	0.0	1.0
1.0	0.0	0.3	0.7	0.8	0.2	0.9	0.1	1	0	0.0	1.0
**0.40**	**0.60**	**0.73**	**0.27**	**0.46**	**0.54**	**0.53**	**0.47**	**0.42**	**0.58**	**0.45**	**0.55**

**Fig. 7:**
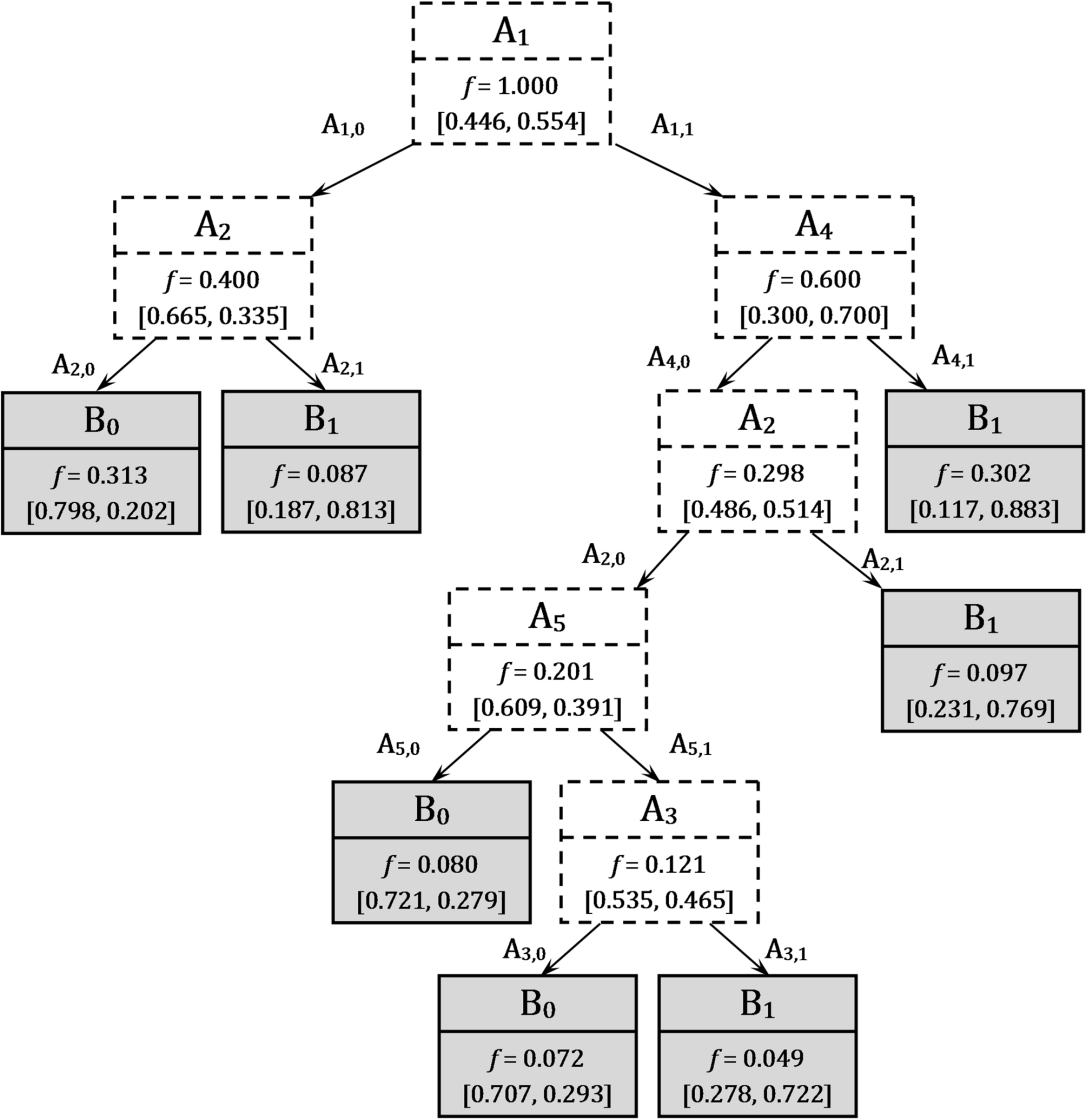
The FDT for the classification of states of the monitoring system.

**Structure Function Construction via FDT** assumes the **FDT induction** as the first sub-stage (Fig. 6). The FDT inducted according to the CMI-based algorithm for the data in Table 5 is in Fig. 7. The leaves in this FDT are indicated in grey. Each leaf has the membership functions of the two possible values B_0_ and B_1_ (last line in leaf image). For example, the first left leaf in Fig. 7 has a value of B_0_ with a confidence of 0.760 and can have a value of B_1_ with a confidence of 0.240. Therefore, the result value is indicated as B_0_. Similarly, the membership functions are specified for the nodes of the input attributes. The possibility of the occurrence of an attribute’s values is defined by its frequency *f*. This value *f* is used for finishing the branch by the leaf if the frequency *f* is less than a defined threshold value α. And the branch is completed by a leaf if the maximum confidence in the decision exceeds the given threshold β. In FDT induction, there are two pruning parameters α and β. The values of α and β are defined from 0 to 1. These values have a direct impact on the FDT size. Fewer values of α and bigger values of β support of FDT induction with the larger size. Large-scale FDT provides a detailed description of training datasets. However, FDT is extremely sensitive to noise in this dataset. The built FDT will have a problem with correctly classifying instances that are not present in the training dataset. The values of thresholds α and β are selected on the training step of the FDT induction.

Please note that the inducted FDT has not been ordered. The attributes at each level of this tree can be different. The CMI-based method for FDT induction in^11^ allows for the development of an ordered FDT. The type of constructed BDD (ordered or no-ordered) depends on the type of the inducted FDT.

The CMI-based algorithm for FDT induction is considered in detail in^11^.

**The transformation to BDD** of inducted FDT is implemented through two procedures: defuzzification and rule-based shortening. An inducted FDT has fuzzy values for output attributes B_0_ and B_1_, which should be transformed into crisp values for reliability analysis. Defuzzification is the process of obtaining a single number from the output of the fuzzy set^24^. The defuzzification can be realised by an ordinary operation, Maximum; this method is known as the *mean-of-maxima* (MOM)^24^. This operation selects the value of output attribute B*j* with the maximal value of membership degree = ((B0), (B_1_)).

**Fig. 8:**
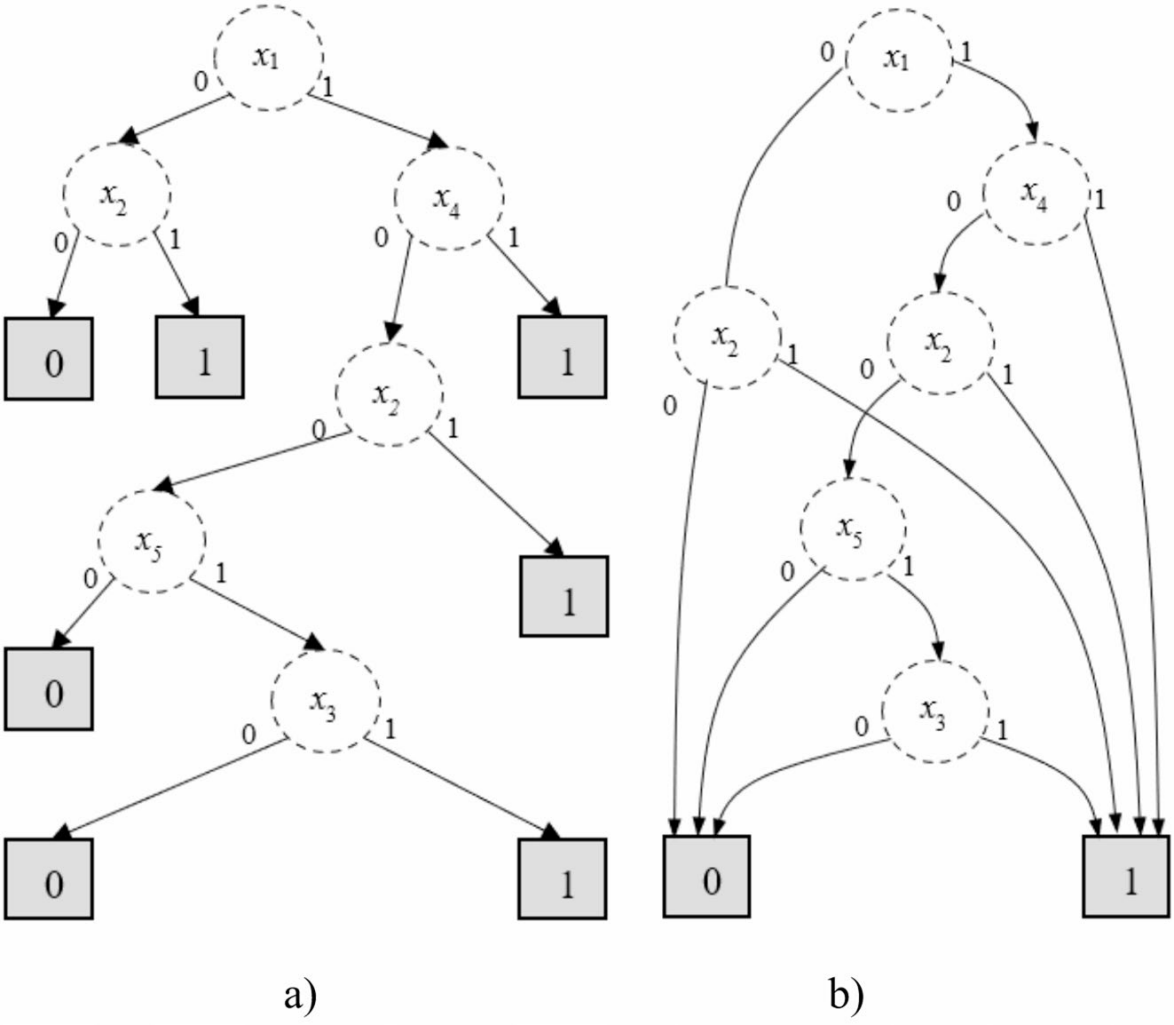
The decision tree (a) and BDD (b) constructed based on the proposed method for the considered monitoring system (Table 5).

For example, the first left leaf after the root of the FDT (Fig. 7) is associated with the output attribute B_0_ because its membership degree is 0.760. The decision tree after defuzzification for the considered example is shown in Fig. 8 (a).

The transformation of the decision tree into BDD is based on three principal rules, similar to those used in BDD construction^18^. Need to be noted that the construction of the optimal/minimal BDD is not the subject of this study. However, inducted FDT is optimal with respect to the number of levels and nodes; therefore, the constructed BDD should also be optimal.

The BDD for the reliability analysis of the monitoring system (Table 5) is illustrated in Fig. 8 (b).

**Mission Availability Analysis** can be computed based on BDD, as shown in Eq. (5). This mission availability is *A* = 0.5473, taking into consideration the probabilities of component states indicated in Table 5. The obtained availability value is quite low, and therefore, it would be advisable to consider improvement scenarios for this mission. According to initial data, the passing of the second segment of this mission has a very high probability of failure (*q*_2_ = 0.73). For example, increasing the probability of successful passing of this segment to *p*_2_ = 0.70 allows the mission availability to *A* = 0.8141. The increase in the probabilities of successful passing of the second and fourth segments to *p*_2_ = *p*_4_ = 0.70 in this monitoring mission allows for improved availability, as *A* = 0.8428. The other possibility, for example, could be a mission performed by two drones instead of one. Based on the observations, it will be necessary to conduct a subsequent analysis and compare the results obtained.

## Evaluation and discussion

The main advantage of the proposed method for analysing drone mission availability is that it relies on well-known, well-tested algorithms (Table 6). Therefore, the effectiveness of the proposed method depends on the algorithms used. Specifically, in this work, we assumed that the source data does not require fuzzyfication and that the success values for each checkpoint or specific path segment are determined by experts with confidence. Failure confidence is defined as 1 minus the success confidence. This interpretation of the source data led to the use of CMI-based algorithms for FDT induction. BDD construction is implemented by MOM defuzzification and the transformation of a decision tree into a BDD^25^. Availability calculation was performed using the algorithms discussed in^12–14,17^.

**Table 6:** The algorithms used in the proposed method.

The proposed method stage	The used algorithm	Reference
**Initial Data Preparation of Drone Mission**	Expert data collection	^24,26^
	Fuzzification	^23,27^
**Structure Function Construction via FDT**		
FDT Induction	Fuzzy ID3	^28^
	Fuzzy CART	^29^
	Cumulative Mutual Information (CMI)-based induction method	^11,13^
Transformation to BDD		
Defuzzification	Mean-of-maximum (MOM)	^25,30^
	Center of Gravity (COG)	^31^
BDD Generation		^18,32,33^
**Mission Availability Analysis**	Availability calculation based on BDD	^12–14,17^

**Fig. 9:**
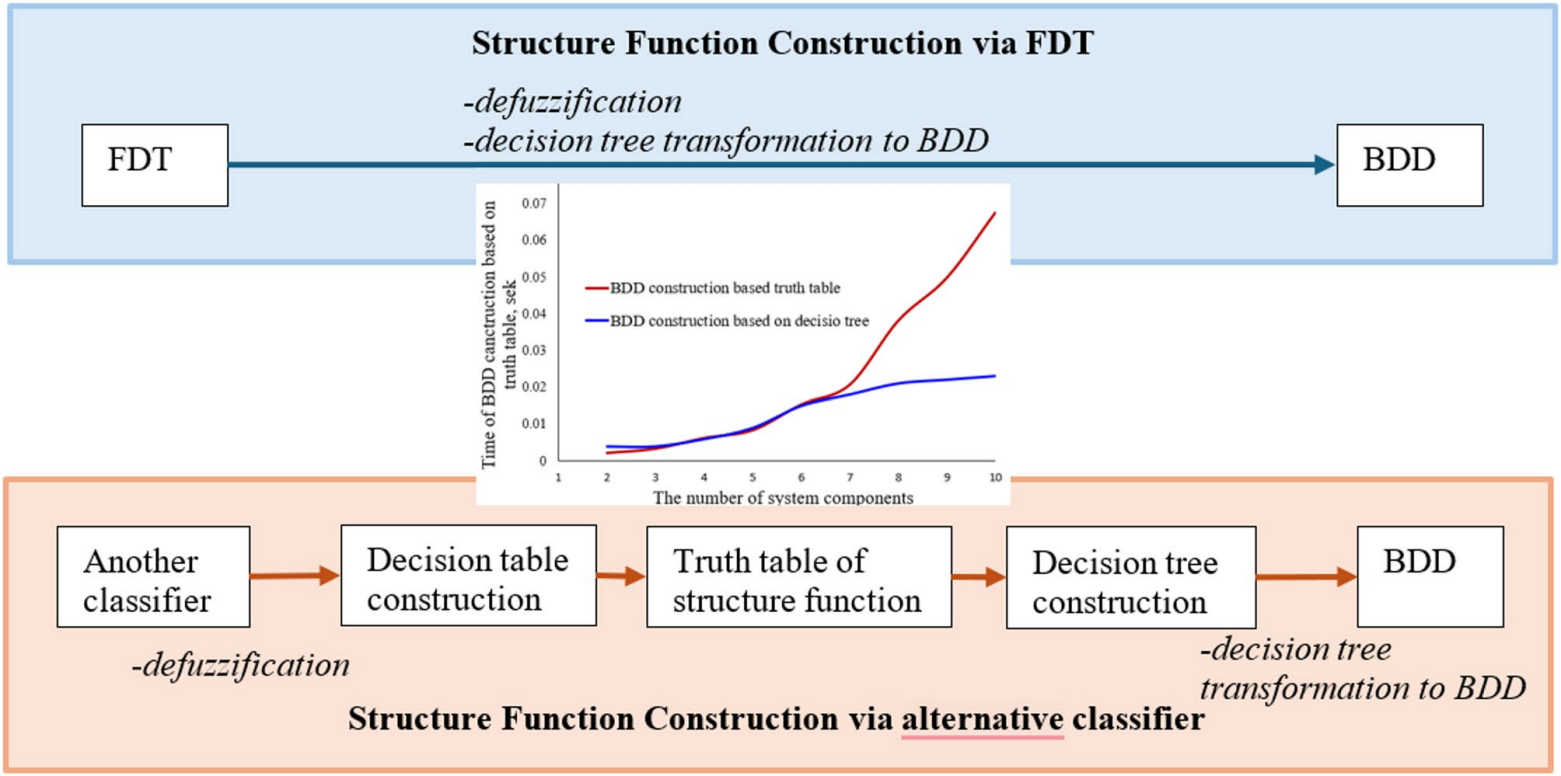
The comparison of the use of FDT and other classifiers in the availability calculation of a drone mission based on BDD.

The algorithms for FDT induction are noteworthy. These algorithms directly impact the accuracy of the resulting model for availability calculation. Nevertheless, among the possible classifiers, we propose the use of FDT, as they have the simplest transformation into a BDD. For example, in the paper^13^, it is shown that the use of another classifier causes an additional step in the transformation FDT - BDD, which is the construction of a decision table, which is interpreted as a truth table of the structure function, and on the basis of which the BDD can then be developed (Fig. 9). According to^13^, the time of BDD construction is increasing exponentially, depending on the number of components of a system, in the case of employing other classifiers due to the construction of a decision table and BDD construction based on a truth table. In the case of the FDT-based classifier, the time of transformation of FDT to BDD is close to a linear change depending on the number of system components.

Figure 9 also provides a qualitative comparison between the proposed FDT‑to‑BDD transformation and alternative uncertainty‑aware classification approaches. While several fuzzy classifiers (e.g., fuzzy CART, fuzzy ID3, fuzzy C4.5) can process uncertain data, they do not produce a tree structure that can be directly transformed into a BDD. Instead, these methods require an additional intermediate step: constructing a complete decision or truth table that represents all possible combinations of component states. For systems with nnn components, this table grows exponentially, which leads to a dramatic increase in computational complexity and memory requirements.

In contrast, the FDT induction algorithm used in our method yields a tree structure that is structurally aligned with a BDD. As illustrated in Fig. 9, this allows for a near‑linear transformation from the induced FDT to the final BDD, without generating an intermediate truth table. This characteristic results in significantly higher scalability when the number of system components increases.

**Fig. 10:**
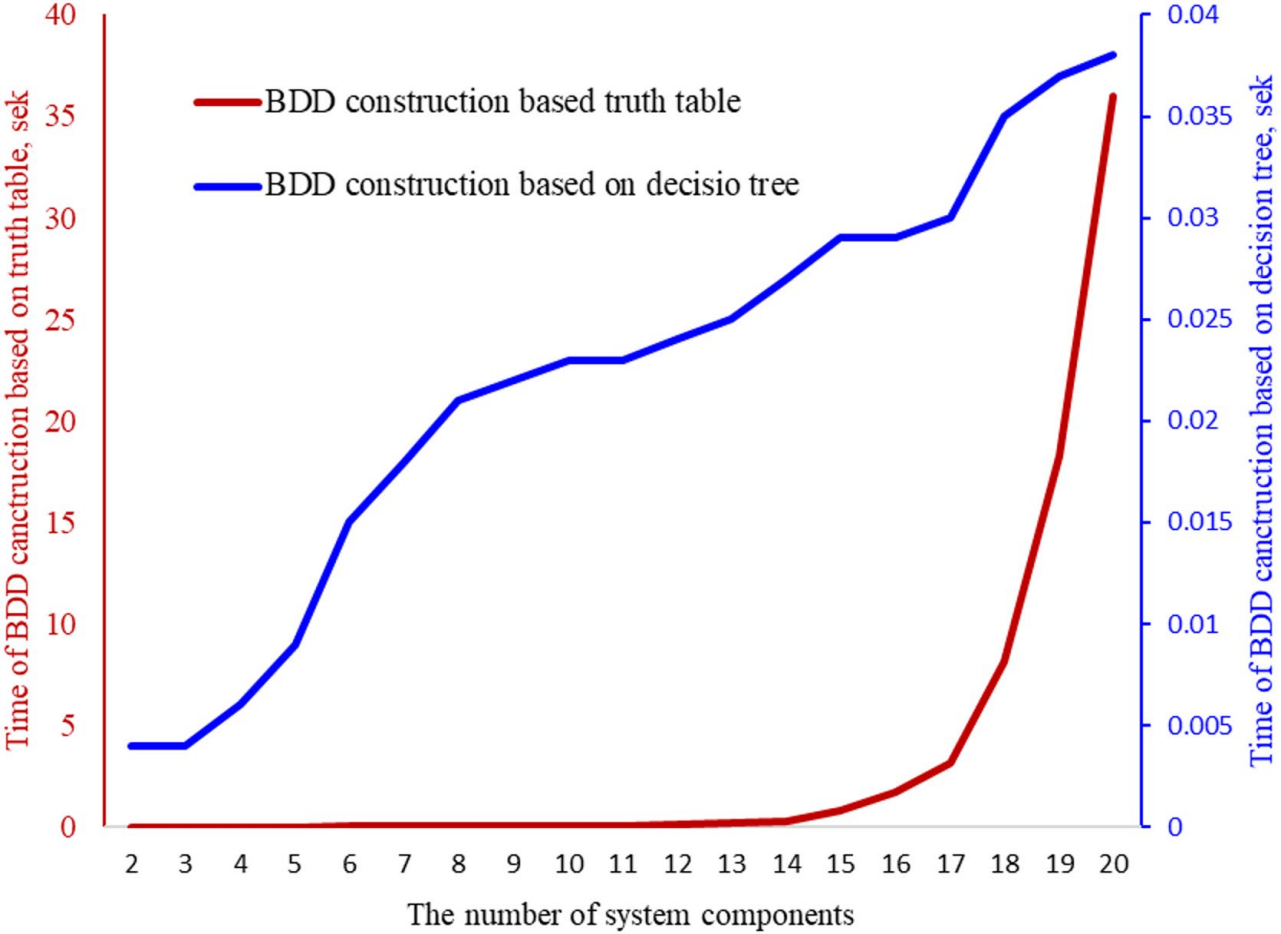
The comparison of time for the BDD construction based on FDT and an alternative classifier.

To highlight this difference, Fig. 10 presents a graphical comparison of the computational effort required by both approaches. The plot uses two vertical axes because the numerical ranges differ significantly. For the proposed FDT–BDD transformation, the values lie within a very narrow interval (approximately 0-0.04), reflecting the low computational cost of the direct transformation. For the alternative classifier–BDD pipeline, the corresponding values range from 0 to 40, which illustrates the significant computational overhead introduced by the mandatory construction of a full decision table prior to BDD generation. The use of separate axes in the figure makes the contrast between the two approaches visually clear and demonstrates the substantial performance advantage of the proposed method. The comparison presented in Fig. 10 shows that the proposed FDT‑based approach avoids combinatorial explosion and offers markedly better computational efficiency than applying alternative classifiers.

Comparing different algorithms for FDT induction reveals that they exhibit similar performance metrics. Table 7 presents the metrics for several algorithms used in FDT induction for the dataset analysed in the forest fire monitoring mission (Sect.  2).

**Table 7:** The Evaluation and Comparison of Algorithms for FDT Induction.

Classification algorithm	F1 Score	Precision	Sensitivity	Specificity	Accuracy
CMI	0.989	0.981	0.991	0.993	0.943
Fuzzy CART	0.963	0.988	0.994	0.995	0.951
Fuzzy C4.5	0.981	0.962	0.989	0.917	0.932
Fuzzy ID3	0.942	0.976	0.982	0.989	0.910

Regarding the FDT induction algorithm, it is also worth noting that, according to the analysis in^11^, which was performed on various datasets, algorithms based on the CMI achieve the highest accuracy when the percentage of given instances falls within the range of 40% to 75%.

The choice of classifier has the greatest impact on the performance of the proposed drone mission analysis method. However, other procedures within the framework also affect the analysis’s effectiveness. In particular, the defuzzification procedure may, in principle, affect the results. In the case of the CMI-based algorithm for FDT induction, the effect of defuzzification on epistemic uncertainty is inherently limited due to both the structure of the input data and the defuzzification rule used.

The membership functions for all input attributes and for the output class are strictly complementary, i.e., $\mu\left({A}_{i,0}\right)+\mu\left({A}_{i,1}\right)=1$ and $\mu\left({B}_{0}\right)+\mu\left({B}_{1}\right)=1$ for every instance. This configuration ensures that the entire epistemic mass is distributed between exactly two mutually exclusive states, with no intermediate or partially overlapping fuzzy values. Under such conditions, defuzzification cannot redistribute or reshape uncertainty; it simply selects the state with the higher support.

We apply the MOM rule, which is one of the least intrusive defuzzification methods. MOM selects the class with the maximal membership degree and does not introduce additional thresholds or weighting assumptions. Due to the complementary nature of the membership degrees, alternative defuzzification strategies (e.g., maximum or centroid) produce identical crisp decisions for all leaves of the FDT. As a result, the crisp decision tree obtained after defuzzification preserves the fuzzy model’s logical structure, and the resulting BDD remains unchanged. For the chosen representation of the initial data, the Centroid method does not affect the BDD construction but has significantly higher computational complexity than MOM. Therefore, although defuzzification is necessary to transform the fuzzy decision tree into a crisp BDD representation, it does not alter the mission success logic or the computed mission availability in our setting.

The proposed method offers a balanced trade-off between modelling capability and practical applicability. Its key strengths and inherent limitations are systematically summarised in Table 7, providing a clear guide for potential users and future research directions (Table 8).

**Table 8:** Advantages and Limitations of the Proposed Method for Drone Mission Analysis.

Aspect	Advantages (Strengths)	Limitations / Future Challenges
Handling of Input Data	The method constructs a formal model (BDD) directly from fuzzy, incomplete, and expert-driven data (confidence degrees), bypassing the need for precise probabilistic data, which is often unavailable in mission planning.	The model’s validity is contingent on the accuracy and consistency of subjective expert evaluations.
Model Interpretability	The resulting BDD provides an intuitive, graphical representation of the mission success logic.	Complexity for very large systems.
Analytical Efficiency	Efficient calculation of reliability metrics. Linear-time transformation from FDT to BDD avoids the combinatorial explosion associated with truth-table-based methods.	Computational cost at the training stage.
Robustness to Data Scarcity	Effective learning from incomplete datasets.	Model accuracy may degrade with extremely sparse datasets (e.g., < 40% of state combinations).
Methodological Background	The method integrates robust, peer-reviewed algorithms for FDT induction and BDD manipulation.	
General Applicability	It shifts reliability analysis from the component (drone) level to the mission success level, aligning directly with operational planning objectives.	Lack of temporal and redundancy analysis.

## Conclusion

In this paper, a novel method for analysing drone mission reliability is proposed. The data for mission analysis can be uncertain and incompletely specified. The proposed method enables the mathematical interpretation and representation of drone missions as BDD, a technique commonly used in reliability analysis. It is also worth noting that there are many BDD-based methods for evaluating system reliability. The undoubted advantage of the considered method is the development of BDD based on incompletely specified and uncertain data. This was made possible by the interpretation of structure function as a classifier and the use of the method for the classifier induction for its construction. There are many methods in machine learning for classifier induction based on incompletely specified data. In particular, for BDD construction, the FDT induction method has been applied. The induction of FDT within the framework of fuzzy logic enables consideration of cognitive uncertainty (ambiguity and vagueness) in the initial data. The choice of the FDT and BDD for the proposed method of drone mission evaluation is motivated by next factors:

- Direct structural compatibility: An FDT is a tree‑based classifier whose internal structure is naturally aligned with the decomposition rules of BDDs. This enables a direct, near‑linear transformation from FDT to BDD without constructing a full truth table, unlike Bayesian networks, probabilistic graphical models, or Markov processes.
- Interpretability: Both FDTs and BDDs provide transparent, human‑readable logic representations. In contrast, Bayesian networks and Markov processes encode system behaviour in joint probability distributions or transition matrices that do not represent mission success logic in a canonical Boolean form.
- Scalability in high‑dimensional Boolean reliability models: BDDs are specifically developed for efficient manipulation of large Boolean reliability structure functions, while Bayesian networks and Markov models suffer from exponential growth in state space or conditional probability tables as the number of mission components increases.
- Computational efficiency: The transformation of FDT to BDD avoids the combinatorial explosion that can occur when transforming from an alternative classifier via the truth table of a structure function (Fig. 9).

For these reasons, the combination of FDTs and BDDs provides a computationally efficient, interpretable, and scalable approach that is particularly well suited for transforming uncertain mission data into a formal reliability model. The proposed method is developed based on studies on constructing a mathematical model from uncertain data^13^ and on reliability analysis of the system using BDD^18^. The application of well-established methods enables its implementation with limited resources. According to a preliminary evaluation, the method can be used for developing a mathematical model of a large-dimensional system comprising 100 components (*n* = 100). It is essential to note that using FDT as the primary classifier enables the creation of a structure function with a sufficiently small number of initial instances, a distinct advantage compared to, for example, neural network-based classifiers.

In this study, the confidence degrees used in the illustrative examples (Sect.  5) represent generic expert-based assessments commonly encountered in mission planning and reliability analysis. The proposed method for drone mission evaluation does not depend on a specific expert-elicitation protocol or on a particular number or type of experts. Instead, it requires only that confidence degrees be available in the standard form of complementary membership values. Such data may originate from any established elicitation process, including structured interviews, consensus-based estimation (e.g., the Delphi method), or aggregation of multiple subjective judgments.

Because the aim of the case study is to demonstrate the computational steps of the methodology rather than to draw conclusions about a particular operational mission, the dataset functions as an example of uncertain or partially specified information. The method remains fully applicable regardless of the number of experts involved, the degree of inter-expert variability, or the specific aggregation rules used to combine expert opinions. When multiple experts are available, their assessments can be aggregated using standard techniques such as averaging, weighted consensus, or normalisation procedures, all of which are compatible with our framework.

Thus, while expert confidence degrees serve as the uncertain inputs in our examples, the validity of the proposed method does not rely on any particular expert group or elicitation protocol, but rather on the general structure of uncertain data that the method is designed to process.

However, it should be noted that this method does not account for the possibility of reservation, which will be one of the next tasks for developing this method and assessing the safety of the drone’s mission^16^. We will also continue this study to evaluate the reliability of drone missions in a time-dependent analysis. This analysis, according to^34^ is possible based on a system representation by a structure function. An important aspect in this future study is the determination of the time-dependent probability of successful checkpoint or path segment passage. In this case, complex observations, depending on various factors and the mission’s execution time, will need to be implemented. Such a process can be unnecessarily time-consuming and expensive. Therefore, we assume the use of a standard probability distribution for each checkpoint or path segment, based on expert estimates (e.g., exponential or Weibull).

Among further research and extensions of the proposed method can be its use in autonomous aerospace systems^35^. Although the present study focuses on reliability‑oriented modelling of UAV missions, uncertainty‑aware decision‑making is also a central theme in autonomous aerospace systems. Prior work in spacecraft navigation and threat avoidance has addressed problems such as debris‑avoidance manoeuvres, autonomous trajectory replanning, and risk‑aware optimal control. These approaches model uncertainty through probabilistic dynamics, Bayesian estimation, Markovian transitions, or reinforcement‑learning‑based policies, and are typically designed for sequential decision-making in continuous time. Future research may extend the proposed method toward more advanced mission‑level risk‑management and decision‑making strategies^36,]^ ^37^. Recent studies in autonomous mission planning have shown the importance of explicitly optimising mission abort decisions, sampling policies, and spare‑resource allocation to control risk throughout a mission. Integrating the structural reliability representation obtained from the BDD into such optimization‑based frameworks would enable the evaluation of not only mission success probability but also mission risk under dynamic operational constraints.

## Data Availability

The datasets used and analysed during the current study are available from the corresponding author upon reasonable request or by link [https://www.dropbox.com/scl/fi/ck0jnl4btcqp675xa22vl/Mission-02.xlsx?rlkey=xe5muebh2trfnygkzqezuutc3&dl=0](https:/www.dropbox.com/scl/fi/ck0jnl4btcqp675xa22vl/Mission-02.xlsx?rlkey=xe5muebh2trfnygkzqezuutc3&dl=0) .
